# Experiences of stigma and discrimination among people experiencing homelessness: a cross-sectional pilot survey in South London, UK

**DOI:** 10.1136/bmjopen-2025-103529

**Published:** 2025-10-20

**Authors:** Andy Guise, Martin McCusker, Jude Adams, River Újhadbor, Simone Helleren, Tuba Mazhari, Lotte Elton, Sujit D Rathod, Lucy Platt

**Affiliations:** 1 King’s College London, London, UK; 2 Lambeth Service Users Council, London, UK; 3 Department of Population Health, London School of Hygiene and Tropical Medicine, London, UK; 4 Department of Social and Environmental Health Research, London School of Hygiene and Tropical Medicine, London, UK

**Keywords:** Health Equity, Health policy, PUBLIC HEALTH

## Abstract

**Abstract:**

**Objectives:**

To understand experiences of stigma and discrimination among adults who are homeless across multiple care and support system contexts.

**Design:**

Cross-sectional survey embedded within an ethnographic case study.

**Setting:**

South London, UK, 2024.

**Participants:**

Convenience sample of 74 people experiencing homelessness, aged over 18 years.

**Results:**

Participants most commonly reported unfair treatment in public settings (85%), legal settings (72%), housing and homelessness services (68%) and health settings (65%). These experiences were attributed to a range of factors and identities, with homelessness the most commonly cited; people commonly linked unfair experiences to multiple identities. People with more comorbidities reported experiencing unfair treatment across more system settings, including and beyond health systems.

**Conclusions:**

Unfair treatment was reported across multiple care and support systems with greater ill-health associated with more unfair treatment. Future larger-scale surveys should measure the extent of stigma and discrimination across the population.

**STRENGTHS AND LIMITATIONS OF THIS STUDY**

- The survey addresses a research gap in quantifying experiences of stigma and discrimination as it relates to homelessness in the UK.
- The survey tool is locally relevant, building on prior work by service user groups, existing scales and then embedded in ongoing ethnographic research in South London.
- The sample size is small, reflecting exploratory research goals (including anticipating the survey tool being a pilot for future research) and pragmatic resource limitations.
- The sample under-represents respondents with English as a second language, who are a significant part of the population of people experiencing homelessness, especially those sleeping rough.

## Background

 The health consequences of homelessness are well documented, in terms of chronic and infectious disease, mental ill-health, injury, violence and drug overdose.^1 2^ Homelessness is growing in the UK. Most recent data for England suggests 242 000 households were experiencing ‘core homelessness’, which includes rough sleeping, hostels, unsuitable temporary accommodation and sofa surfing, with this figure having risen since 2020.^3^ Policy debate often focuses on rough sleeping: this too has risen, with over 3000 people seen rough sleeping in 2022, again up on previous years (ibid).

Stigma refers to how some people, conditions or places are marked out as undesirable or degraded,^4^,^6^ and discrimination is the differential treatment of the stigmatised.^7^ Stigma is widely recognised as a core challenge with respect to homelessness^8 9^ and especially with reference to how stigma shapes health and healthcare experiences for people experiencing homelessness.^10 11^ The field of homelessness and stigma is, though arguably, under-conceptualised^12^ and has not generated focused attention on measuring specific experiences of stigma and discrimination (e.g. Kidd, 2007).^13^ Available studies do explore how experiences of stigmatisation among people experiencing homelessness are linked to poor health, through impacting on mental health and well-being, and also through limiting healthcare access.^10^ A substantial qualitative literature, including in the UK, on homelessness and health has also shown how stigma is embedded in ongoing challenges across multiple systems and settings.^14 15^

This study aims to address a gap in research in quantifying where people experiencing homelessness in the UK experience stigma and discrimination, and then to explore associations with health and well-being. In the context of growing homelessness in the UK, it is important to develop this in-depth understanding of the role and consequences of stigma and discrimination.

We report here on a cross-sectional study of experiences of stigma and discrimination among people experiencing homelessness in South London. The study was primarily intended to address specific research goals of informing an ongoing ethnographic study and policy goals of fostering debate in South London services around the nature of stigma and discrimination. The study also secondarily functioned as a pilot study to assess feasibility for large-scale implementation of the questionnaire in and beyond London.

## Methods

### Design

We implemented a cross-sectional survey to measure experiences of stigma and discrimination among people experiencing homelessness in South London. The survey was co-produced through a partnership between academic researchers and representatives of a drug and alcohol service users council from South London. The survey was embedded within an ethnographic case study of how stigma and discrimination relating to homelessness is experienced and comes about in South London; the survey was part of a mixed-methods design, where the instrument and approach were based on qualitative work and consultation, and then the survey results are the basis for ongoing qualitative study.

### Setting

The survey was conducted in three boroughs in South London, UK. Lambeth, Lewisham and Southwark are central districts with large populations of people experiencing homelessness. Most recent figures for 2023–2024^16^ are that over 1600 individuals were seen sleeping rough across the three boroughs. Of these people, approximately 40% were British, 20% from the European Union, and then significant minorities (ie, over 5%) of people from Africa, Asia and the Americas; further, of those identifying as British, a large proportion are black British (ranging from 10–29% across the three boroughs), whether identifying as African or Caribbean (in this way this region of South London is distinct from other areas of London and the UK); people recorded in official monitoring of rough sleeping are mainly men (over 80%) (CHAIN, 2024).

### Survey implementation

We implemented the survey in service settings focused on the needs of people experiencing homelessness, including two hostels, three day centres and one drug and alcohol service, across the three London boroughs of Southwark, Lambeth and Lewisham from June to November 2023; sites were selected based on their ongoing involvement in the ethnographic study in which the survey was embedded. Management at each site was asked for permission to implement the survey. We did not implement the survey in street settings to ensure that support to people completing the survey was available (eg, if someone became distressed, or an urgent need became apparent).

The survey was completed in English on paper, either self-completed or administered by a researcher who would guide respondents to the questions, read aloud where necessary and complete the relevant fields. The respondent had the choice of method of completion. We supported access to the survey through having it available in braille, larger fonts to allow for vision impairment and on yellow paper to account for dyslexia. The survey was anonymous. After completion, surveys were stored securely and then manually inputted to Excel, with the majority double-checked to ensure accuracy. Data was held on a secure network.

### Participants

We used a convenience approach to sampling, appropriate given the exploratory aims of the survey,^17^ that in turn built on the resource challenges of operationalising a random or quota driven approach to sampling. While not seeking to be representative in a statistical sense, we sought to purposefully ensure a range of respondents to allow for subgroup analysis. Our initial target was to recruit a minimum of 150 people, considered feasible within resource constraints and research experience of the team. As described below, the survey took longer to administer given emotional issues it generated and time taken to complete, and so we limited our sample within those constraints.

People were eligible if they were currently homeless, following expansive definitions as used in National Health Service and UK Government approaches^18 19^ which include rough sleeping; being a temporary resident in hostels, night shelters, safe houses and unsupported temporary accommodation; or temporarily staying with family and friends. People also needed to be over the age of 18, and currently residing in and/or using services focused on the needs of people experiencing homelessness in South London. We approached participants within the service settings and asked them to complete the questionnaire; we chose times of late in the morning or early afternoon to be most convenient, and this within the conventional opening hours of each service. We did not monitor numbers of people potentially eligible to participate nor reasons for non-participation to allow calculation of a response rate, given the pilot status of the study and the contingencies of the settings where this would have been invasive and interrupted the ongoing services or support people were getting.

### Measures of stigma and discrimination

The survey was co-produced through a partnership between researchers in a drug and alcohol service users network, the Lambeth Service Users Council (LSUC), and university-based researchers at King’s College London (KCL). The LSUC implemented a survey in 2018 of experiences of stigma among people using drug and alcohol services in the London Borough of Lambeth.^20^ This survey found high levels of stigma across different services and domains of society. The 2018 survey was influential within services and led to specific policy efforts around language and inclusion. A critique of the LSUC survey from some was that it was not being sufficiently ‘academic’; while important, such critiques are also problematic, potentially reflecting less the rigour of research and instead hierarchies of knowledge, and so stigma. However, reflection in 2022 by the LSUC and KCL teams led us to use the 2018 LSUC survey as a foundation for developing a new questionnaire built on academic literatures that would explore how people experience stigma across multiple different systems and domains of society. We integrated approaches from academic research, in particular (1) integrating questions and scales on stigma,^1321^,^23^ (2) developing the survey to allow for statistical analysis and (3) using academic protocols around ethical review. The revised survey therefore sought to be more ‘academic’, in terms of being conceptualised with reference to broader literatures, but we emphasise here we do not understand there to be a binary of ‘academic’ and ‘community’ research. We also developed the survey based on ongoing ethnographic fieldwork and consultations with people experiencing homelessness. See online supplemental file for the full survey instrument. The measures we report in this paper are experiences of discrimination and their attributions, current health conditions and demographic characteristics, detailed below.

#### Experiences of discrimination across multiple system domains and their attributions

Our questions about experiences of stigma and discrimination built on the *Major Experiences of Discrimination Scale* and *Everyday Experiences of Discrimination Scale* as adapted to the UK and mental health by Gabbidon *et al*.^21^ Gabbidon *et al* use questions to explore the experience of discrimination—defined above as the differential treatment of the stigmatised—following the formulation of having experienced ‘unfair treatment’ and relate this to different settings. Respondents are then asked to attribute these experiences to demographic and social categories; such categories being what in our study we see as potentially stigmatised.

We developed a list of settings where people might report unfair treatment. We borrowed from the list of Gabbidon *et al* and combined this with the system settings from the original LSUC survey, settings from other scales used to understand mental health and drug use stigma,^22 24^ our ethnographic data and consultations with people experiencing homelessness. We identified 18 different settings where people experiencing homelessness could experience unfair treatment. Respondents were then asked to respond to a series of statements relating to each system setting, for example, ‘I have been unfairly treated at the GP’, with options of yes, no, not sure, not applicable. For analysis, we also grouped these individual system and public settings into eight broader domains: housing, health, drug and alcohol services, legal services, welfare, public settings, work and education (not all of the domains including multiple services and settings, eg, drug and alcohol services).

Following the approaches of the scales listed, we also asked participants what they attributed any unfair treatment to.^21^ Gabbidon *et al*^21^ sought attributions for each area of discrimination. Such precision is time-consuming, given the 18 different settings we were exploring for potential discrimination, and we sought a short survey. We followed Scheim and Bauer,^23^ who ask about experiences of discrimination and then ask once for people to attribute this to particular factors. We built on the list of demographics and social characteristics from Gabbidon *et al*, Scheim and Bauer, and then from our consultations and ethnographic fieldwork to identify 19 characteristics from which people could select, with an option to self-describe if preferred. We were wary of the survey framing stigma as a function of the individual rather than a social process and so prefaced this question with: ‘Stigma can be attached to many different experiences and things. If stigma is attached to something, we don’t think that is right. Also, any stigma is about a problem in society and not the individual that might be impacted by it (eg, the problem is racism, not race)’.

#### Health conditions

We asked people to report any current health conditions. We used a list from a survey recently implemented in London to understand health service access among people experiencing homelessness.^25^ We adjusted language and added items based on consultations as above.

#### Demographic characteristics

We asked people to state their age, gender, whether they currently used drugs or alcohol and to self-describe what they use. We asked about ethnicity using UK Office for National Statistics categories^26^ as widely used in surveys on discrimination in the UK; we did however adapt the wording slightly, taking out reference to ‘any other’ which is included if the existing categories do not apply. Use of ‘other’ we saw as resonating with the concept of ‘othering’ which is a core component of stigma and discrimination^6^; consultations on the draft survey confirmed this. We instead included options for people to use another term to describe their background if that was useful.

The draft survey instrument was discussed and piloted with stakeholders across South London, including with people with experience of homelessness. While we built on validated scales, our adaptations to them were not formally validated. Instead, we sought to develop a survey that was locally relevant. Any future scaled-up implementation of the survey would then involve more formal validation.

### Statistical analyses

Statistical analyses were performed using Stata (V.18). Summary statistics were calculated for demographic data. The analyses presented here are descriptive statistics.

In reporting experiences of unfair treatment, we created binary variables to distinguish those who had experienced stigma from those who had not in each domain; we report the number of people and then proportion. Individuals could report what they attributed unfair treatment to (we report both how many people reported each attribute and then the number of attributes reported by individuals was totalled).

To explore the relationship between experiences of unfair treatment and health, we created mutually exclusive groups to distinguish individuals by how many of the eight domains they reported unfair treatment in (as above); these two groups were 1–3 domains and then 4–8 domains. We then explored the relationship between the number of domains unfair treatment was reported in and two measures of health. First, was comorbidity status; the number of health issues was totalled for each individual, and we created categories of none, one, two or three or more health issues. We report frequencies for each category. Second, was type of health issue experienced. We created health categories: neurodiversity and learning difficulty (attention deficit hyperactivity disorder, dyslexia, autism), mental health issues (psychosis, depression, bipolar, mental health); HIV and hepatitis C virus, issues relating to teeth, cardiovascular disease or risk factors for cardiovascular disease (issues with the heart, diabetes or high blood pressure), breathing issues (asthma, chronic breathing problems, chronic obstructive pulmonary disease, tuberculosis) and drugs or alcohol use. Binary variables were created to distinguish those who did and did not have each of these health issues. We report frequencies for each category.

All missing data were excluded from analysis; where data on specific questions are missing, they were coded as such and reported where relevant.

Results were discussed within selected homeless hostels and day centres in South London, to refine analysis and to identify potential interpretations.

### Monitoring implementation

As above, the study had a secondary aim to pilot a survey in this context to assess feasibility for future larger-scale implementation of the survey. We had regular reflective discussions as a team through implementation, leading to the identification of lessons for future development and for immediate adaptations.

### Patient and public involvement

People with experience of homelessness and related experiences of drug use were involved in the design of the study, development of measures, and then engaged in recruitment and analysis of data. This involvement happened through formal partnerships and then through individual meetings with members of the public, for example, to discuss the draft survey.

Each participant was given £10 compensation for their time.

## Results

The survey was completed by 74 people, although two responses were not included in the final analysis: 2 people were recruited in error (they had reported being homeless through recruitment, but then in the final questions reported they were currently housed).

### Reflections on piloting

Implementation of the survey generated findings to inform future development. We adapted the survey through implementation after recognising the emotional consequences of completing the survey for some participants. Many welcomed the chance to discuss the issues, as reported to us in open-text responses at the end of the survey asking for their views on the survey. Some also directly reported, or it became apparent through their reactions, that discussing past or ongoing experiences of stigma and discrimination was upsetting. As per our ethical review and safeguarding protocols, in such situations, we would pause and offer to end the survey, and then discuss any issues arising, and make any links to appropriate staff. We also adapted the survey during implementation to lessen the emotional toll: one set of questions asked for an estimate of ‘how many times’ particular forms of unfair treatment had happened. It became clear that asking respondents to specify a number of times was requiring them to mentally count through, and so remember, all past experiences; we changed this to the binary of ‘once/more than once’ to allow quicker responses based on an intuition rather than requiring participants to consider all potentially traumatic experiences. While we had assumed most people would prefer to self-complete the survey, the vast majority of people chose to complete with a member of the research team. While we never asked for a reason, people often offered the reason of eye-sight and not having glasses with them. Literacy may have been a factor in some situations. The intuition of the research team was also that people preferred to discuss such sensitive topics, rather than have to manage this alone. No one completed the survey using the versions in braille, larger font or on yellow paper to support those with dyslexia; we did not have a clear protocol for asking consistently about peoples’ preferences for versions with larger font or yellow paper which may partly explain this. We had originally planned to have the survey translated into Portuguese, Spanish and Polish, reflecting the significant populations in South London experiencing homelessness who primarily speak these languages. However, we cancelled this plan through implementation. Members of the research team with these languages were no longer available and given the potential emotional toll of completing the survey—as above—we did not want people self-completing a survey and then not being able to discuss any issues arising with the research team.

Table 1 summarises the principal demographic characteristics of those surveyed. Experiences of homelessness varied, with 24 respondents in hostels or supported accommodation, 16 sofa surfing and 12 sleeping rough. Most respondents were aged 51–60 (n=23) and a majority were men (n=61).

**Table 1:** Demographic characteristics of respondents, South London, 2024

Characteristics	Total (n)	Percentage (%)
Sociodemographic factors	n	%
Age		
20–30	4	6
31–40	17	24
41–50	17	24
51–60	23	32
61–70	10	14
Missing	1	1
Gender		
Male	61	85
Female	9	12
Missing	2	3
Ethnicity		
Asian	2	3
Black	11	15
Mixed	5	7
Additional ethnic group	1	1
White	48	67
Missing	5	7
Sexual Orientation		
Heterosexual/straight	59	82
Gay/lesbian	3	4
Bisexual	3	4
Do not know	1	1
Prefer not to say	2	3
Prefer to self-describe	1	1
Missing	3	4
Drug use		
Yes	38	54
No	29	42
Prefer not to say	2	3
Missing	3	4
Alcohol use		
Yes	45	63
No	25	35
Prefer not to say	0	0
Missing	2	3
Where did you sleep last night?		
Hostel/supported accommodation	24	33
Sleeping on somebody’s sofa	16	22
Sleeping rough	12	17
Other	15	21
Prefer not to say	2	3
Missing	3	4

Table 2 summarises where people reported ‘being unfairly treated’ across 18 different services or settings, grouped also into the six domains (where people reported unfair treatment in any of the subcategories). People principally reported being unfairly treated in public settings (85%), legal settings (72%), then housing and homelessness settings (n=49, 68% of respondents) and healthcare (n=47, 65%).

**Table 2:** Reported unfair treatment across service and system settings

Service or setting, grouped into domains	Number of people reporting unfair treatment	Proportion of people reporting unfair treatment
	n	%
**(1) Housing**	**49**	**68**
Housing services	46	64
Hostel, day centre or refuge	24	33
**(2) Health**	**47**	**65**
GP	29	40
Dentists	17	24
Pharmacy	13	18
Hospital	29	40
Mental healthcare	24	33
**(3) Drug and alcohol services**	**13**	**18**
**(4) Legal**	**52**	**72**
Police	48	67
Court	38	53
**(5) Welfare**	**38**	**53**
**(6) Public settings**	**61**	**85**
Shops	41	57
Public transport	31	43
Pubs/café/restaurant	24	33
Social media	11	15
Family	31	43
**(7) Work**	**27**	**38**
**(8) Education**	**20**	**28**

GP, General Practice .

Table 3 reports how many people selected each of the listed experiences or identities as contributing to experiences of unfair treatment, with people being able to select more than one. Housing and homelessness was reported the most (68%), followed by poverty (46%), drug use (43%), welfare (43%) and mental health (40%).

**Table 3:** Experiences and identities that unfair treatment was attributed to

N=72	n	%
Housing	49	68
Poverty	33	46
Drug use	31	43
Welfare	31	43
Mental health	29	40
Alcohol use	25	35
Ethnicity	22	31
Physical health	20	28
Disability	19	26
Age	12	17
Citizenship	11	15
Gender	10	14
Sexual orientation	8	11
Other	8	11
Parenthood	6	8
HIV	5	7
Sex work	4	6
Hepatitis C	3	4

Table 4 reports the frequency of the total number of experiences or identities reported as contributing to unfair treatment. The majority of participants (76%) attributed the unfair treatment they had experienced to at least two experiences or identities, with many participants attributing them to four, five, six or more.

**Table 4:** Number of intersecting attributions for unfair treatment experienced

Attributions	n	%
0	8	11
1	7	10
2	12	17
3	6	8
4	7	10
5	8	11
6	3	4
7	8	11
8	4	6
9	3	4
10	2	3
11	1	1
12	1	1
Missing	2	3

Table 5 summarises the relationship between the number of system domains of stigma—as in table 2 above—and then health status, as defined by (1) number of health issues and then (2) type of health issue, with groupings based on health issues commonly associated with experiences of homelessness.

**Table 5:** Relationship of health issues with unfair treatment across system domains

	n	Number of system domains in which stigma was reported	
		1–3	4–8
		Row frequency (%)	Row frequency (%)
(1) Number of health issues			
None	4	2 (50%)	2 (50%)
One	7	4 (57%)	3 (42%)
Two	14	10 (71%)	3 (21%)
Three or more	47	8 (17%)	37 (78%)
(2) Type of health issues			
Neurodiversity and learning difficulty	17	3 (18%)	13 (76)
Psychosis, depression, bipolar disorder	49	10 (20%)	38 (78%)
HIV and hepatitis C	4	2 (50%)	2 (50%)
Teeth	33	10 (30%)	23 (70%)
Heart problems, high blood pressure, diabetes	19	4 (21%)	14 (74%)
Asthma, chronic breathing problems, COPD, TB	22	7 (32%)	14 (63%)
Drug and alcohol use	28	6 (21%)	22 (79%)

COPD, chronic obstructive pulmonary disease; TB, tuberculosis.

People reporting three or more health issues reported the highest frequency of unfair treatment: of the 47 people with three or more health issues, 37 people reported unfair treatment across 4–8 system domains. People most commonly reported mental health, teeth and drug and alcohol as health issues, and of these, 78%, 70 and 79%, respectively, reported unfair treatment across 4–8 system domains.

## Discussion

Through a survey of people experiencing homelessness in South London, 74 people reported their experiences across different systems of care and support. Before discussing the analysis of experiences of unfair treatment and health, we first discuss what we learnt from the process and the implications for future surveys.

The measures and process we developed were generally acceptable to the people surveyed and brought insight to experiences of stigma across multiple different system settings, although a number of lessons have been identified for future implementation. Additional resourcing for staffing and refining of protocols would address issues of language access. Additional steps could be taken to lower the potential of upsetting participants and support access. We noted that participants often wanted to discuss their experiences in more depth, which in our overall study design was facilitated through follow-up qualitative interviews at a later date, although we often had long discussions with participants while completing the survey that were not recorded as data. Other designs could be prioritised in future, as for example, those used in mental health focused literatures on stigma^24^ that integrate qualitative responses within quantitative survey questions. However, the emotional experiences we identified through the survey may not be a function of the specific methodology, and instead point to particular aspects of the experiences being studied. Being currently homeless as compared with other experiences might be more associated with isolation from reliable forms of social support and a greater number of intersections with other ongoing hardships and traumas; completing a survey in this life context may be more likely to be challenging. Furthermore, the experience of the survey may also, or instead, reveal the social, political and economic context of the UK in the study period 2023–2024: of the realisation of the consequences of diminished funding for services^27^ on top of a longer-running UK housing crisis.^28^ Our survey implementation may then be revealing the despair that emerges from particular political and economic conditions. Paying more attention to how the experience of completing a survey is embedded within the experience of the hardships being studied would be essential in future iterations of the survey.

The analysis of what people reported led to three principal findings. First, people most commonly reported unfair treatment across multiple system settings. Second, these experiences were attributed to a range of factors and identities, with homelessness the most commonly cited. Third, people with more comorbidities report experiencing unfair treatment across more system settings. We discuss each finding in turn. As noted, and we return to below, the study sample size is small and any implications of these findings we are cautious on.

Unfair treatment is reported in high levels across multiple systems and settings. Literatures on healthcare and homelessness have long noted poor experiences in these settings.^10^ Similarly, long histories of criminalisation of homelessness^29^ also correspond to the high proportion of respondents reporting negative experiences in legal settings, including the police and the courts system. We note here that the UK recently repealed 200-year-old legislation that criminalised rough sleeping; such efforts could reduce such experiences of unfair treatment in the future, although local authorities in the UK also use other legislation to criminalise behaviour they would previously have used the vagrancy act for.^30^ That housing and homelessness services were very high is itself a novel finding. Further research is needed here and will be a focus for our ongoing ethnography. This ethnography should explore whether, for example, unfair treatment with respect to housing and homelessness services is an ongoing series of minor incidents and erosion of status as compared with policing and healthcare that might be less frequent but more traumatic, for example, violence or denial of care. As suggested by respondents in analysis workshops, an unfair experience within housing and homelessness services may also be inevitable given the length of time people are in them and how they can be experienced as hostile and negative spaces.^31^

Our second finding focuses on the range of experiences and identities attributed to unfair treatment. Given the increasing recognition of intersectional experiences of discrimination,^23^ this finding corresponds to existing literature, including as it relates to homelessness.^32^,^34^ In the context of the preceding analysis that has detailed the range of system settings in which people experience stigma, this finding alerts us to the overall potential complexity of a systemic understanding of stigma: intersecting systems of disadvantage are combining and being mediated through multiple care and support systems. More analytical attention is needed to understand these potential systemic properties. Future survey research with larger samples could explore more what particular clusters of experiences of intersectional stigma there are, to alert to particular systemic forms of stigma involved.

That people with more health issues report stigma across more domains of society could partly reflect how people are engaged in more services and so there are more opportunities in which stigma can arise. However, this logic would not clearly explain why stigma is experienced across domains beyond health. This finding therefore also points to particular facets of the experience of stigma, for example, why more health experiences might engender stigma within the context of legal or housing systems or in use of public transport. As above, future larger scale surveys should explore these clusters of experiences more, including associations with particular health conditions, whereby the role of particular conditions, which may or may not be visible or indicated or be potentially ‘hidden’ may activate particular stereotypes.

### Limitations

Our survey and the findings explored here have significant limitations, most notably the small convenience sample, even if participants in the study had a range of experiences and approximately correspond to many dimensions of the population of South London who are homeless. Our study sample was not large enough to allow for detailed exploration of how particular experiences varied or intersected, for example, by gender or race and ethnicity as they relate to particular experience of homelessness. Given these limitations, the findings here should be cautiously interpreted in how they describe experiences among people experiencing homelessness in South London; consequently, their generalisability to other settings beyond South London is also limited. However, the study nonetheless gives important insight to the range of settings in which people experiencing homelessness experience unfair treatment.

## Conclusions

The survey we implemented among people experiencing homelessness in South London, UK has identified important aspects of stigma and discrimination: of being experienced across multiple care and support systems, involving multiple intersections of experiences and identity, and that health comorbidities are associated with experiencing stigma across different care and support systems, not just healthcare. Future larger-scale surveys should explore more the particular intersections of experiences indicated here, and in turn foster analytical and policy attention to the multiple systems involved in stigmatising people experiencing homelessness.

## Supplementary material

10.1136/bmjopen-2025-103529 online supplemental file 1

## Data Availability

No data are available.
