# Supplementary material for: Experiences of stigma and discrimination among people experiencing homelessness: a cross-sectional pilot survey in South London, UK
Source: BMJ Open. 2025 Oct 20;15(10):e103529. doi: 10.1136/bmjopen-2025-103529 (PMC12542552; doi:10.1136/bmjopen-2025-103529)
Supplement: online supplemental file 1 [file bmjopen-15-10-s001.docx]

**ARE YOU**


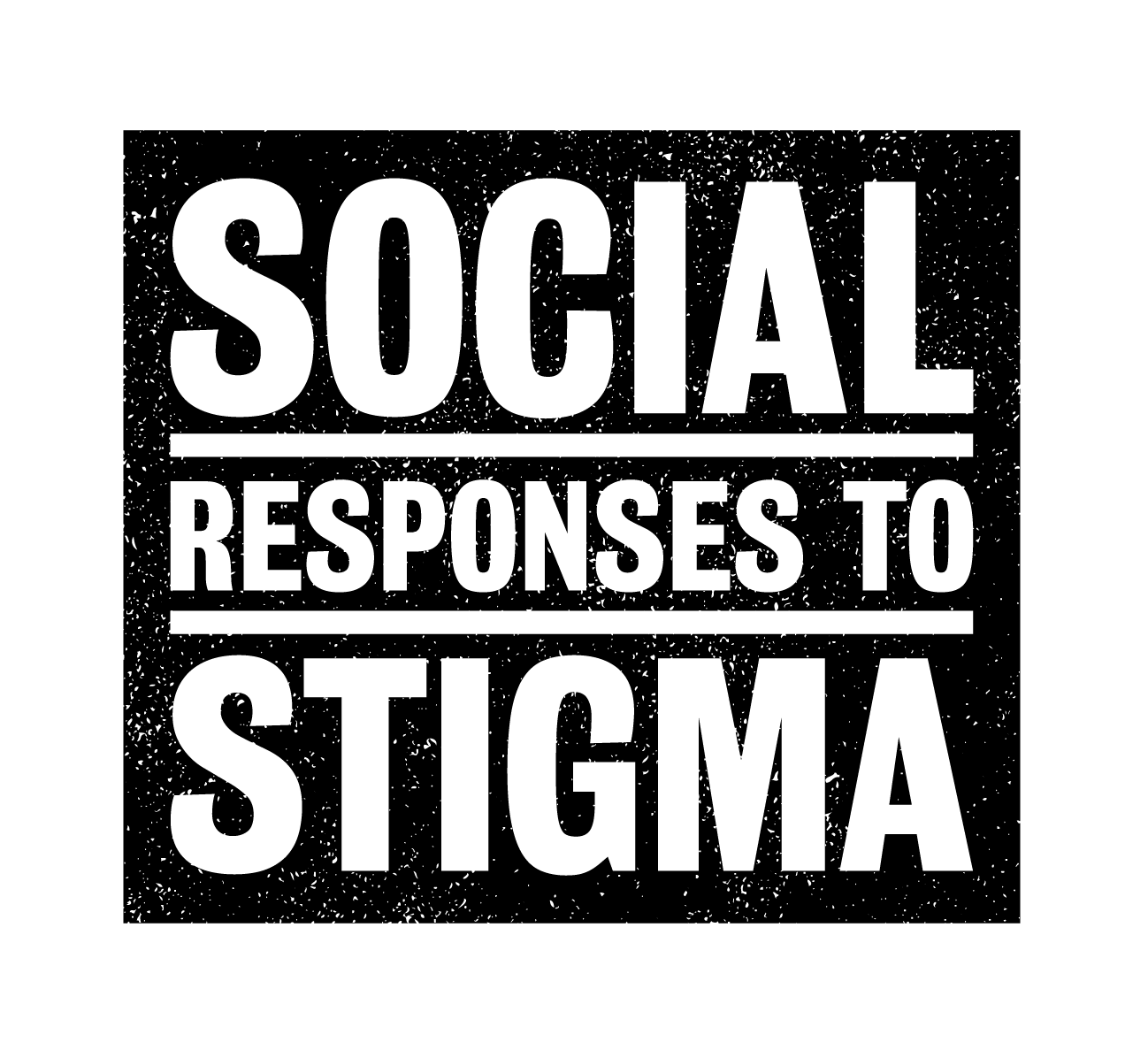


**TREATED**

**FAIRLY?**

A survey of experiences of health care, welfare, housing and other services you might use.


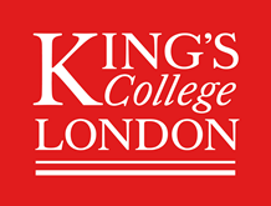

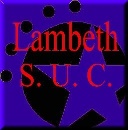


**What is this survey about?**

We are doing a survey of experiences of various forms of care and support across south London.

We are interested in whether people feel stigmatised and discriminated against, or not.

The study aims to improve care and support systems.

The survey is being implemented by a team of researchers from King’s College London and the Lambeth Service Users Council, also working with the 999 club.

The questions in the survey are about whether you have experienced unfair treatment or not, where this was, what you think caused it, and what impacts you think this has had for you. We then ask a few basic questions about you, such as your age, your ethnicity and about your health.

**The survey is anonymous**. We won’t ask for your name or contact details.

**It will take about 10 to 15 minutes to complete.**

As a thank you for your time we will give you £10 cash/voucher.

The survey is confidential. The only time we might talk to someone else about what you say is if you tell us that you or someone else is in danger. If this happened, we would stop the survey and talk to you, and then talk to someone else who could help.

**The survey is voluntary.** You do not have to take part in the survey. If you do not participate this will have no impact on your use of any services.

If you do participate you do not have to answer any question you don’t want to or you can stop the survey at any time, and you don’t need to give a reason.

We know that talking about experiences of unfair treatment can be difficult. If you think you will get upset thinking or talking about this we would advise you not to complete the survey.

A member of our research team will sit with you to help fill the survey in. Or, if you prefer, you can fill the survey in yourself (we need you to do this with a member of the team nearby though, in case you have any questions).

If you have any access needs please tell us. We have the survey in other languages – Polish, Portuguese and Spanish – and different formats that might help.

After the survey we will give you more information about participating in other parts of our study, including an in-depth interview.

You should ask the person implementing the survey if you have any questions. They will be able to answer your questions and provide more information.

Do you have any questions?

If yes, please discuss with the research team until you are happy.

If you are interested to complete the survey, we have 3 quick questions to check you are eligible:

1 are you aged over 18?

2 do you consider yourself to be currently homeless? By this we mean anyone who is sleeping rough, living in temporary accommodation, temporarily staying with family or friends or any other inadequate or insecure accommodation.

3 is this the first time you have completed the survey?

If you answered yes to these questions you are eligible to complete the survey.

By continuing to fill in the survey you understand that:

1 your participation is voluntary, and you understand what the survey is about.

2 you don’t have to answer any question you don’t want to, and you can stop the survey at any time without a reason.

3 you don’t need to give your name and everything is confidential. The only time a researcher might talk to someone else about what is said is if you say that you or someone else is in danger.

If you understand these three points and would like to still complete the survey the questions begin on the next page.

If you would prefer not to complete the survey please stop here. We are very grateful for your time and interest in the survey.

| ***Survey administration*** | |
| --- | --- |
| A1 | Anonymous reference number …………. |
| A2 | Initials of research team member ………….. |
| A3 | What Borough are you currently completing the survey in? Select one by circling it  1 Lambeth  2 Lewisham  3 Southwark |
| A4 | Where are you completing the survey? Select one:  1 Hostel or other temporary or sheltered accommodation  2 Day centre  3 Outreach or street setting  4 Over the phone  5 Prefer to self describe……………………………………………………………………………. |

| ***Your experiences of stigma and discrimination*** | |
| --- | --- |
|  | There are lots of definitions of stigma. We understand stigma as when people are seen by wider society as ‘marked’ and different, and that this is a bad thing. This stigma can then lead to people being treated unfairly (which we can also call discrimination). **Does that definition make sense to you?** This survey will be asking about your experiences in relation to these ideas. |
|  | In the next few questions we would like to ask about experiences or feelings you might have had. **As a reminder, you don’t have to answer any questions you don’t want to, and you can stop the survey at any time.** |

| B1 | **Anticipating Stigma**  The next questions are about whether you ever worry about being seen differently and treated unfairly in different areas of your life. |  |
| --- | --- | --- |
|  | \| I sometimes or always worry that I will be \|  \| \| --- \| --- \| \| \| 1 unfairly treated by a GP \| Yes / no / not sure / not applicable \| \| 2 unfairly treated in a hospital \| Yes / no / not sure / not applicable \| \| 3 unfairly treated when seeking or receiving mental health care \| Yes / no / not sure / not applicable \| \| 4 unfairly treated by the police \| Yes / no / not sure / not applicable \| \| 5 unfairly treated at work \| Yes / no / not sure / not applicable \| \| 6 unfairly treated in the education system \| Yes / no / not sure / not applicable \| \| 7 unfairly treated in the court system \| Yes / no / not sure / not applicable \| \| 8 unfairly treated when using public transport \| Yes / no / not sure / not applicable \| \| 9 unfairly treated by dentists \| Yes / no / not sure / not applicable \| \| 10 unfairly treated by housing services \| Yes / no / not sure / not applicable \| \| 11 unfairly treated in a hostel, day centre or refuge \| Yes / no / not sure / not applicable \| \| 12 unfairly treated when seeking welfare benefits \| Yes / no / not sure / not applicable \| \| 13 unfairly treated online or on social media \| Yes / no / not sure / not applicable \| \| 14 unfairly treated in a shop \| Yes / no / not sure / not applicable \| \| 15 unfairly treated by family \| Yes / no / not sure / not applicable \| \| 16 unfairly treated in a drug and alcohol service \| Yes / no / not sure / not applicable \| \| 17 unfairly treated in a pharmacy \| Yes / no / not sure / not applicable \| \| 18 unfairly treated in a pub, café or restaurant \| Yes / no / not sure / not applicable \| |  |
| B2 | **Experiencing stigma and discrimination**  The next questions are about how you have been treated in different areas of your life.   \| I have been \|  \|  \| How often in your life?  Please estimate number of times \| Was this ever in the borough where you are completing the survey?  Please circle \| \| --- \| --- \| --- \| --- \| --- \| \| 1 unfairly treated by a GP \| Yes  No  Not sure  Not applicable \| If yes \|  \| Ever  Never  Not sure \| \| 2 unfairly treated in a hospital \| Yes  No  Not sure  Not applicable \| If yes \|  \| Ever  Never  Not sure \| \| 3 unfairly treated when seeking or receiving mental health care \| Yes  No  Not sure  Not applicable \| If yes \|  \| Ever  Never  Not sure \| \| 4 unfairly treated by the police \| Yes  No  Not sure  Not applicable \| If yes \|  \| Ever  Never  Not sure \| \| 5 unfairly treated at work \| Yes  No  Not sure  Not applicable \| If yes \|  \| Ever  Never  Not sure \| \| 6 unfairly treated in the education system \| Yes  No  Not sure  Not applicable \| If yes \|  \| Ever  Never  Not sure \| \| 7 unfairly treated in the court system \| Yes  No  Not sure  Not applicable \| If yes \|  \| Ever  Never  Not sure \| \| 8 unfairly treated when using public transport \| Yes  No  Not sure  Not applicable \| If yes \|  \| Ever  Never  Not sure \| \| 9 unfairly treated by dentists \| Yes  No  Not sure  Not applicable \| If yes \|  \| Ever  Never  Not sure \| | |
|  | B2 continued   \| I have been \|  \|  \| How often in your life?  Please estimate number of times \| Was this ever in the borough where you are completing the survey?  Please circle \| \| --- \| --- \| --- \| --- \| --- \| \| 10 unfairly treated by housing services \| Yes  No  Not sure  Not applicable \| If yes \|  \| Ever  Never  Not sure \| \| 11 unfairly treated in a hostel, day centre or refuge \| Yes  No  Not sure  Not applicable \| If yes \|  \| Ever  Never  Not sure \| \| 12 unfairly treated when seeking welfare benefits \| Yes  No  Not sure  Not applicable \| If yes \|  \| Ever  Never  Not sure \| \| 13 unfairly treated online or on social media \| Yes  No  Not sure  Not applicable \| If yes \|  \| Ever  Never  Not sure \| \| 14 unfairly treated in a shop \| Yes  No  Not sure  Not applicable \| If yes \|  \| Ever  Never  Not sure \| \| 15 unfairly treated by family \| Yes  No  Not sure  Not applicable \| If yes \|  \| Ever  Never  Not sure \| \| 16 unfairly treated in a drug and alcohol service \| Yes  No  Not sure  Not applicable \| If yes \|  \| Ever  Never  Not sure \| \| 17 unfairly treated in a pharmacy \| Yes  No  Not sure  Not applicable \| If yes \|  \| Ever  Never  Not sure \| \| 18 unfairly treated in a pub, café or restaurant \| Yes  No  Not sure  Not applicable \| If yes \|  \| Ever  Never  Not sure \|   If you answered no/not sure/not applicable to all of the above please go to question C1. | |

| B3 | If you answered yes to any of the options in the last question (B2), what do you think might have contributed to it?  Stigma can be attached to many different experiences and things. If stigma is attached to something we don’t think that is right. Also, any stigma is about a problem in society and not the individual that might be impacted by it (e.g. the problem is racism, not race).  Please circle **as many things** from the list below that you think might have contributed to you being unfairly treated.  Stigma attached to:  1 Age  2 Gender  3 Self identity as trans or gender non-binary  4 Sexual orientation  5 Ethnic origin or race  6 Religious affiliation  7 Disability  8 Housing status  9 Mental health  10 Physical health  11 HIV status  12 Hep C status  13 Drug use  14 Alcohol use  15 Poverty  16 Use of welfare benefits  17 Sex work  18 Citizenship or migration status  19 Parenthood  20 Prefer to self-describe - please specify…………………………………………………. |
| --- | --- |

| B4 | If you answered yes to any of the items in B2 which **one** has had the most impact on you?  1 unfairly treated by a GP  2 unfairly treated in a hospital  3 unfairly treated when seeking or receiving mental health care  4 unfairly treated by the police  5 unfairly treated at work  6 unfairly treated in the education system  7 unfairly treated in the court system  8 unfairly treated when using public transport  9 unfairly treated by dentists  10 unfairly treated by housing services  11 unfairly treated in a hostel, day centre or refuge  12 unfairly treated when seeking welfare benefits  13 unfairly treated online or on social media  14 unfairly treated in a shop  15 unfairly treated by my family  16 unfairly treated by drug and alcohol services  17 unfairly treated in a pharmacy  18 unfairly treated in a pub, café or restaurant |
| --- | --- |
| B5 | If you answered yes to anything in B2 do you think it made your physical health worse?  Yes / no / not sure / not applicable |
| B6 | If you answered yes to anything in B2 do you think it made your mental health worse?  Yes / no / not sure / not applicable |
| B7 | if you answered yes to anything in B2 do you think your experiences have been worse since the start of the COVID-19 pandemic?  Yes / no / not sure / not applicable |

| B8 | Have you avoided services because of how you have been treated?  Yes  No  Not sure  If yes, which service…………………………………………………………………………………………….. |
| --- | --- |
| B9 | Have you avoided services that you haven’t been to before because you worry you will be unfairly treated?  Yes  No  Not sure  If yes, which service…………………………………………………………………………………………….. |

| **Complaining about stigma** | |
| --- | --- |
| C1 | Have you ever made a formal complaint about an experience of unfair treatment?  Yes / no / not sure / not applicable  (if yes, skip to C3) |
| C2 | Do you know how to make a formal complaint about an experience of unfair treatment?  Yes / no / not sure |
| C3 | Would you feel comfortable telling a person who is treating you unfairly that they should stop?  Yes / no / not sure |
| C4 | Do you know anyone who can help you when you feel treated unfairly?  Yes / no / not sure |

| **Some information about you** | |
| --- | --- |
| In this final section of the survey we are asking some questions about you to help understand how experiences of stigma might vary for different people  As a reminder: the survey is anonymous. We don’t want to know your name or contact details.  You don’t have to answer any questions you don’t want to.  We know that these questions can feel a bit like being ‘put in a box’ and make people feel uncomfortable. We are asking them though so that we can understand the different experiences people have. | |
| D1 | What age are you?  ……………………………………………………………………………………………….  Prefer not to say |
| D2 | What gender are you?  1 Male  2 Female  3 Prefer not to say  4 Prefer to self describe………………………………………………………………….. |
| D3 | Is your gender identity the same as the sex you were assigned at birth?  1 Yes  2 No  3 Prefer not to say |

| D4 | **What is your ethnic group?**  Choose one option that best describes your ethnic group or background  **White**  1. English/Welsh/Scottish/Northern Irish/British 2. Irish 3. Gypsy or Irish Traveller 4. If another term is useful to describe your white background please describe…………………………………………………………………………………………………..  **Mixed/Multiple ethnic groups**  5. White and Black Caribbean 6. White and Black African 7. White and Asian 8. If another term is useful to describe your Mixed/Multiple ethnic background, please describe…………………………………………………………………………  **Asian/Asian British**  9. Indian 10. Pakistani 11. Bangladeshi 12. Chinese 13. If another term is useful to describe your Asian background, please describe…………………………………………………………………………………………………..  **Black/ African/Caribbean/Black British**  14. African 15. Caribbean 16. If another term is useful to describe your Black/African/ Caribbean background, please describe……………………………………………………  **Additional ethnic group**  17. Arab 18. If another term is useful to describe your ethnic group, please describe………………………………………………………………………………………………………………… |
| --- | --- |

| D5 | Do you drink alcohol?  Yes / no / prefer not to say  If yes, what are you drinking and how much per day on average? |
| --- | --- |
| D6 | Do you use drugs?  Yes / no / prefer not to say  if yes, what is your drug of choice, and how do you use it? |

| D7 | Do you currently have any of the following health problems or conditions? Circle all that apply, or go to the next question if you prefer not to say   \| 1 ADHD \| \| --- \| \| 2 Asthma \| \| 3 Autism \| \| 4 Brain injury \| \| 5 Bipolar disorder \| \| 6 Cancer \| \| 7 Chronic breathing problems (bronchitis, emphysema, obstructive airways disease) \| \| 8 Depression or anxiety \| \| 9 Diabetes \| \| 10 Difficulty seeing / eye problems \| \| 11 Drug (addiction) problems \| \| 12 Dyslexia \| \| 13 Epilepsy / seizures \| \| 14 Foot problems \| \| 15 Heart problems (including angina, murmur, abnormal rhythm) \| \| 16 Hepatitis C \| \| 17 High blood pressure \| \| 18 HIV \| \| 19 Joint, bone or muscle problems \| \| 20 Long COVID \| \| 21 Psychosis \| \| 22 PTSD \| \| 23 Sexually transmitted infection (chlamydia, gonorrhoea or pelvic inflammatory disease) \| \| 24 Skin/wound infection \| \| 25 Stomach problems \| \| 26 Teeth / dental problems \| \| 27 Tuberculosis \| \| 28 Additional physical or mental health issues, please describe………………………… ……………………………………….. \| |
| --- | --- | --- | --- | --- | --- | --- | --- | --- | --- | --- | --- | --- | --- | --- | --- | --- | --- | --- | --- | --- | --- | --- | --- | --- | --- | --- | --- | --- | --- |

| D8 | Which of the following best describes how you think of yourself?  1 Heterosexual/straight  2 Gay/lesbian  3 Bi  4 Don’t know  5 Prefer not to say  6 Prefer to self-describe…………………………………………………………………………. |
| --- | --- |
| D9 | In your life have you ever – circle all that apply  1 Stayed with friends, relatives or other people because you had no home of your own  2 Stayed in a hostel, foyer, refuge, night shelter or b&b hotel because you had no home of your own  3 Slept rough  4 Squatted  5 Applied to the council as homeless or as threatened with homelessness  6 Spent time in local authority care as a child  7 Begged (that is, asked passers-by for money in the street or another public place)  8 Shoplifted because you needed things like food, drugs, alcohol or money for somewhere to stay  9 Prefer not to say |
| D10 | How old were you when you first became homeless?  As a reminder – for this survey, by homelessness we mean anyone who is sleeping rough, living in temporary accommodation, temporarily staying with family or friends or any other  1………………………………………………………………………………………………………………..  2 Prefer not to say |
| D11 | How long ago did you last have secure housing?  1………………………………………………………………………………………………………………..  2 Prefer not to say |

| D12 | Where did you sleep last night?  1 Sleeping rough on streets/parks  2 In a hostel or supported accommodation  3 Squatting  4 Sleeping on somebody’s sofa/floor  5 In emergency accommodation – e.g. night shelter/refuge  6 In B&B or other temporary accommodation  7 In a hospital  8 Public transport  9 Prefer to self describe…………………………………………………………………………………….  10 Prefer not to say |
| --- | --- |
| D13 | Was where you slept last night in this borough?  1 Yes  2 No  3 Not sure  4 Prefer not to say |
| D14 | Are you a citizen of  1 The UK  2 European Union  3 Another country  4 Prefer not to say |
| D15 | Are you eligible to receive state benefits in the UK?  Yes / no / not sure / prefer not to say |

| **Closing** | |
| --- | --- |
| E1 | Is there anything else you would like to tell us or think is important about stigma and discrimination? The researcher might also write anything here that helps explain any responses to questions you have already made. |
| E2 | Do you have any feedback on the survey? Were there any questions that you didn’t like or were hard to answer, or questions that you were glad we asked? |
|  | **This is the end of the survey.** |

| **More information** | |
| --- | --- |
|  | If you would like us to talk with you about any of the issues raised by this survey please just ask the research team. A member of the research team will also give you a copy of The Pavement magazine which has lots of resources and contacts in which may also be helpful.  **Is there anything you would like to talk about?** |
|  | This survey you have just completed is one part of our study on stigma. If you would be interested to talk to the research team about your experiences and views in more depth they can give you information about doing an in-depth interview. |
|  | If you would like to know about the results of the study and to attend feedback events you can find out more information on our website, see posters in the hostels in the borough in the months to come, or, we can note down your contact details separately to contact you (as the survey is anonymous, we would note down your contact phone number, address or email in a separate system, completely unlinked to the information you have just given us). |
|  | **Thank you for filling out the survey.** |
