# Supplementary material for: Experiences of stigma and discrimination among people experiencing homelessness: a cross-sectional pilot survey in South London, UK
Source: BMJ Open. 2025 Oct 20;15(10):e103529. doi: 10.1136/bmjopen-2025-103529 (PMC12542552; doi:10.1136/bmjopen-2025-103529)
Supplement: Supplementary file 2 [file bmjopen-2025-103529.reviewer_comments.pdf]

PEER REVIEW HISTORY

BMJ Open publishes all reviews undertaken for accepted manuscripts. Reviewers are asked to complete a checklist review form and are provided with free text boxes to elaborate on their assessment. These free text comments are reproduced below.

ARTICLE DETAILS

Title (Provisional)

Experiences of stigma and discrimination amongst people experiencing homeless: a cross-sectional pilot survey in south London, UK

Authors

Guise, Andy; McCusker, Martin; Adams , Jude; Ujhadbor, River; Helleren, Simone; Mazhari, Tuba; Elton, Lotte; Rathod, Sujit D; Platt, Lucy

---

VERSION 1 - REVIEW

Reviewer 1

Name Mabhala, Mzwandile

Affiliation University of Chester Faculty of Health and Social Care, Department of Public Health and Wellbeing

Date 04-May-2025

COI None

This article focuses on a critical social and health issue: homelessness. The research is carried out ethically. However, one area that could be improved is the engagement with the literature that supports the main concepts discussed in the article.

The opportunity to engage effectively with literature on three key concepts—discrimination, stigma, and homelessness—has been missed in this article. The authors imply that individuals experiencing homelessness face stigma and discrimination due to their situation; however, they fail to present literature that argues that discrimination and stigma are significant causes of homelessness.

1. At a minimum, the article should have defined the concepts of discrimination and stigma in relation to its context and referenced the work of key authors on these topics.

2. In lines 4 to 6, ONS provides official UK statistics on homelessness. It is unclear why the authors did not reference this source, particularly given the notable omission of a large, established researcher in the UK's homelessness field.

3. In lines 9 to 11, I do not understand how this sentence contributes to the main argument regarding stigma, discrimination, and homelessness.

4. Lines 14 to 16 would have more effectively supported the core arguments if they had been phrased as "stigma and discrimination are widely recognized as determining factors in homelessness through multiple pathways."

5. Lines 16 to 18 are vague. Are the authors suggesting that stigma is a primary barrier to accessing healthcare for individuals experiencing homelessness?

Methods

6. Line 3 makes reference to CHAIN, this needs to be spelt out in full
7. Authors report that of 40% of people experiencing homelessness, the significant majority were black-British; about discrimination, authors appear uncritical of this fact.

Participants

8. Line 28 sentence begins with "We used a convenience approach…. Please include references.
9. Line 32 states, "Our initial aspirational target was to recruit 150 people …. Please explain how you decided on this aspirational target.
10. Line 34 states, "As described above, the survey took longer to implement given emotional and time needs." What do the authors mean by this?
11. Line 37 talks about UK Government definitions… please provide the definition and reference.

Measures of stigma and discrimination

12. Please provide a reference to the Lamberth Service Users Council survey.

Discussion

13. Page 7, lines 41 to 60, could come later in the discussion.

Reviewer 2

Name Perrotta, Carla

Affiliation University College Dublin, School of Public Health, Physiotherapy and Sport Sciences

Date 15-May-2025

COI None

This study addresses a highly relevant and topical question:"What are the experiences of stigma and discrimination amongst people experiencing homelessness..."

Overall, the manuscript is well-written and well-structured. I was interested in your study and engaged, which nowadays tells you something!

I have comments regarding the survey methodology, data collection, and statistical analysis that the authors could clarify/review.

Survey:

The methodology of how the survey was adapted and/or developed needs clarification. What specific constructs related to stigma and discrimination were measured? How were these constructs operationalised? Did you conduct ny validation processes (e.g., piloting, cognitive testing, psychometric assessment)? If no validation was undertaken, the rational needs to be specified in the text. In the case that this study is actually a validation of the survey as well, then it will need to be clearly defined in the objectives.

Survey administration

Did the interviewer converse with the participant, guiding them from one question to another, and then complete the relevant fields? Or did the participant read and complete the survey themselves?

Statistical analysis

Using descriptive statistics and cross-tabulations appears appropriate for the study's aims. I recommend omitting references to regression analysis. Planning a regression analysis with the sample size/covariates/multiple categories per variable would not have been appropriate.

Discussion

The first section of the discussion focuses heavily on the tool's limitations. If survey development /validation is a key contribution of this study, it should be explicitly stated as an objective in the introduction and methods. If not, this section may be better positioned under limitations.

Additionally, the discussion would benefit from a short paragraph addressing interactions with the judiciary system, as participants identified this as an area of systemic neglect. Further contextualisation or reference to existing literature could strengthen this section.

In conclusion

This valuable and timely study highlights systemic issues faced by people experiencing homelessness. The authors should provide more detail on survey development, clarify data

collection procedures, and refine/adjust the discussion. I look forward to seeing these revisions in a future version of the manuscript.

**Reviewer**	**3**
**Name**	**Currie, Jane**
**Affiliation**	**Queensland University of Technology, School of Nursing**
**Date**	**15-May-2025**
**COI**	**None**

Thank you for your submission, I read it with fascination. The paper is generally well written. The methods are especially well described in a high level of detail. I was pleased to read the thoughtful approach of the research team in adapting the questionnaire during the study in response to participants experiences of completing it.

When I read the results I wondered whether educational attainment and literacy had any bearing on perceived stigma, i.e., not finishing school, not gaining GCSE's and A-levels or equivalent.

Also, in describing homelessness, I tend to use the term 'people experiencing homelessness' which tends to be less stigmatising by inferring the potential temporary nature of homelessness - it is something i am experiencing but not something I am.

Best of luck with this work.

VERSION 1 - AUTHOR RESPONSE

Reviewer: 1
Dr. Mzwandile Mabhala, University of Chester Faculty of Health and Social Care
Comments to the Author:

This article focuses on a critical social and health issue: homelessness. The research is carried out ethically. However, one area that could be improved is the engagement with the literature that supports the main concepts discussed in the article.

The opportunity to engage effectively with literature on three key concepts— discrimination, stigma, and homelessness—has been missed in this article. The authors imply that individuals experiencing homelessness face stigma and discrimination due to their situation; however, they fail to present literature that argues that discrimination and stigma are significant causes of homelessness. We agree that the critical engagement with the literature in the first draft was limited. There are gaps you have suggested below which we have tried to address. One factor in this limited critical engagement is word count. We would like to include a discussion on how stigma and discrimination are significant *causes* of homelessness, with this being important context. However, a discussion of stigma as a cause of homelessness necessarily involves exploring multiple axes of discrimination (on the basis that discrimination relating to

experiences other than homelessness is what precedes being homeless). Given that this would be context and isn't directly relevant to the study question – how stigma and discrimination is experienced when homeless – we thought it necessary to leave out.

1. At a minimum, the article should have defined the concepts of discrimination and stigma in relation to its context and referenced the work of key authors on these topics. We agree, and this was an oversight in the first draft. We have included definitions and core citations for these.

2. In lines 4 to 6, ONS provides official UK statistics on homelessness. It is unclear why the authors did not reference this source, particularly given the notable omission of a large, established researcher in the UK's homelessness field. We used the statistics from the Homeless Monitor (see Fitzpatrick et al citation) as the measures here are considered by many to be more comprehensive. Whilst the ONS does provide official statistics they are accepted by many in the sector as an underestimate.

3. In lines 9 to 11, I do not understand how this sentence contributes to the main argument regarding stigma, discrimination, and homelessness. The sentence in the first draft was written as: "*The health consequences of homelessness are well documented, in terms of chronic and infectious disease, mental ill-health, injury, violence and drug overdose (Hwang and Burns, 2014, Aldridge et al., 2017)*." Our intention here was to frame homelessness as a direct challenge for health, and so as necessary for consideration by the journal readership. The ordering in the paragraph wasn't though clear, with the sentence not clearly flowing on from the previous statistics. We have reordered the paragraph so that this sentence is first.

4. Lines 14 to 16 would have more effectively supported the core arguments if they had been phrased as "stigma and discrimination are widely recognized as determining factors in homelessness through multiple pathways." We agree that the sentence as previously written was unclear. On reviewing the manuscript we decided that the idea wasn't essential to include, as our goal with the sentence was to provide broader context around stigma and health. This no longer seemed necessary in light of the definitions included after your comments and the overall need to be clear and concise.

5. Lines 16 to 18 are vague. Are the authors suggesting that stigma is a primary barrier to accessing healthcare for individuals experiencing homelessness? We were suggesting that and we have rewritten the sentence to try and increase clarity.

Methods

6. Line 3 makes reference to CHAIN, this needs to be spelt out in full – The use of the acronym that was unexplained was unclear. We have deleted the acronym seeing as the detail is already in the reference list.

7. Authors report that of 40% of people experiencing homelessness, the significant majority were black-British; about discrimination, authors appear uncritical of this fact. We would be grateful if you could clarify your comment. We noted in the manuscript that 'a large proportion' of people experiencing homelessness are Black-British. In hindsight, this was unhelpfully vague. Across the three boroughs studied the percentage of people who are rough sleeping that report their identity as Black British (including African, Caribbean and Other options together) is in a range from 10% to 29%. We have included that in the text to clarify it.

With respect to discrimination and appearing uncritical – we aren't clear on your precise meaning. We have considered this in relation to three possible points:

- Data in the 'setting' section of the methods: we haven't said more in the 'setting' text as there aren't data relating to race, homelessness and discrimination that we are aware of (indeed, this was one of the logics for the study).
- Results: race and ethnicity were attributes that people could report as being what they attributed any unfair treatment to – in our results we reference the most commonly reported, and race and ethnicity (not just relating to being British) was reported by 31% of respondents as a factor. We could report more sub-analyses in the manuscript – e.g. what those who identified as Black-British attributed any unfair treatment to. A challenge here is the already limited sample for the analysis, and how there are a series of sub-analyses that would logically need doing given the populations of the south London boroughs. We acknowledge this is an important analysis and will report it in other forms locally – e.g. reports, workshops.
- Discussion: we note in the limitations of the paper that given the sample size we were unable to understand some experiences in-depth, and we noted gender here as an example. Given the data cited about race in south London it is also clearly important that this is prioritised in future, and so we have also referenced that as an example.

Participants

8. Line 28 sentence begins with "We used a convenience approach…. Please include references. – We have included a reference to this paper - *dos Santos, M. M. L., et al. (2014). "An exploratory survey measuring stigma and discrimination experienced by people living with HIV/AIDS in South Africa: the People Living with HIV Stigma Index." BMC Public Health **14**(1): 80.* We are grateful for the reminder of a reference here, as this citation had been omitted through the various drafts, and we consider it important to include for how it shaped our overall thinking on the survey design: of using pragmatic judgements, including convenience sampling, when proportionate to a research goal

9. Line 32 states, "Our initial aspirational target was to recruit 150 people ….Please explain how you decided on this aspirational target. This was based on a judgement of what was feasible within the research context. We have amended the language in the relevant sentence to reflect the role for this judgement (rather than, for example, calculations based on assumptions of study power and confidence level): "Our initial target was to recruit a minimum of 150 people, considered feasible within resource constraints and research experience of the team." Please note – we added 'minimum' to clarify the nature of the target, and deleted 'aspirational' as we now don't consider the language to be very clear.

10. Line 34 states, "As described above, the survey took longer to implement given emotional and time needs." What do the authors mean by this? This was an error. We should have said 'below', referring to the opening section of the results where we talk about the process of implementing the survey and how that took unexpectedly long.

11. Line 37 talks about UK Government definitions… please provide the definition and reference. Again, our error, we have now included this.

Measures of stigma and discrimination

12. Please provide a reference to the Lamberth Service Users Council survey. This has now been included.

Discussion

13. Page 7, lines 41 to 60, could come later in the discussion. We considered this. Having the discussion on the process of implementation after the discussion of specific findings would be a useful logic in terms of fitting a general convention of 'theory then practice' which is intuitively clear. However, given the results section starts with the description of implementation we have continued with the current ordering of the discussion so that the logical flow between the results and discussion is clear. We are happy to reconsider this if you think essential to move.

Reviewer: 2
Dr. Carla Perrotta, University College Dublin
Comments to the Author:
This study addresses a highly relevant and topical question:"What are the experiences of stigma and discrimination amongst people experiencing homelessness..."

Overall, the manuscript is well-written and well-structured. I was interested in your study and engaged, which nowadays tells you something!

I have comments regarding the survey methodology, data collection, and statistical analysis that the authors could clarify/review.

Survey:
The methodology of how the survey was adapted and/or developed needs clarification. What specific constructs related to stigma and discrimination were measured? How were these constructs operationalised? Did you conduct any validation processes (e.g., piloting, cognitive testing, psychometric assessment)? If no validation was undertaken, the rational needs to be specified in the text. In the case that this study is actually a validation of the survey as well, then it will need to be clearly defined in the objectives. We consider these linked questions in two steps. First to address the points on which constructs were explored and how they were operationalised, second to address the points on validation.

Constructs and their operationalisation – we have included detail to clarify the core constructs of i) discrimination and then ii) what that is attributed to.

Validation – we did not undertake extensive validation processes. We built on and adapted validated scales and questions (Gabbidon et al, Scheim and Bauer – two core references cited in the text). We did conduct pilots and discussed and explored the questions with stakeholders, including with people with current and past experience of homelessness. We didn't undertake validation beyond this, e.g. cognitive testing or psychometric assessment. This approach reflecting our survey had limited resources and that we sought to explore a priority policy issue in the south London context; the survey was also intended to develop a potential methodology for future use, and our intention is that in any future scale-up we would seek further validation. We have included detail to this effect in the text.

Survey administration
Did the interviewer converse with the participant, guiding them from one question to another,

and then complete the relevant fields? Or did the participant read and complete the survey themselves? We have included text to clarify that both methods of completion were available to respondents for them to choose.

Statistical analysis
Using descriptive statistics and cross-tabulations appears appropriate for the study's aims. I recommend omitting references to regression analysis. Planning a regression analysis with the sample size/covariates/multiple categories per variable would not have been appropriate. We have omitted these references; we had included them to foster transparency in how the study evolved, but agree that this is unnecessary and distracts.

Discussion
The first section of the discussion focuses heavily on the tool's limitations. If survey development /validation is a key contribution of this study, it should be explicitly stated as an objective in the introduction and methods. If not, this section may be better positioned under limitations. As above, we did seek to develop a survey for this setting and for future widespread implementation, although didn't seek extensive formal validation. The introduction includes a brief statement of this development and piloting as a secondary aim, and then we also added a brief discussion in the methods of how we monitored implementation – briefly, through regular reflective discussions as a team to identify lessons and immediate adaptations needed.

Additionally, the discussion would benefit from a short paragraph addressing interactions with the judiciary system, as participants identified this as an area of systemic neglect. Further contextualisation or reference to existing literature could strengthen this section. We agree and have added some detail noting the context for this of the criminalisation of homelessness in specific UK legislation, and noted how that legislation has been repealed, which may lead to improvements.

In conclusion
This valuable and timely study highlights systemic issues faced by people experiencing homelessness. The authors should provide more detail on survey development, clarify data collection procedures, and refine/adjust the discussion. I look forward to seeing these revisions in a future version of the manuscript.

Reviewer: 3
Dr. Jane Currie, Queensland University of Technology
Comments to the Author:

Thank you for your submission, I read it with fascination. The paper is generally well written. The methods are especially well described in a high level of detail. I was pleased to read the thoughtful approach of the research team in adapting the questionnaire during the study in response to participants experiences of completing it.

When I read the results I wondered whether educational attainment and literacy had any bearing on perceived stigma, i.e., not finishing school, not gaining GCSE's and A-levels or equivalent. This would be another plausible factor that stigma could be attributed to. This

wasn't raised in the discussions that shaped the survey, and is a point we will consider for future development of the survey. A related point we have considered is that of literacy – a factor for many people in their experience of education and which is overly represented amongst experiences of homelessness; again, this didn't emerge in our development of the tool. In future work we will consider these together.

Also, in describing homelessness, I tend to use the term 'people experiencing homelessness' which tends to be less stigmatising by inferring the potential temporary nature of homelessness - it is something i am experiencing but not something I am. Thank you – we had been inconsistent on this terminology, using 'experiencing' often, but also frequently using 'who are'. We have changed this to your suggestion.

VERSION 2 - REVIEW

**Reviewer**	**2**
**Name**	**Perrotta, Carla**
**Affiliation**	**University College Dublin, School of Public Health, Physiotherapy and Sport Sciences**
**Date**	**16-Jul-2025**
**COI**	

Thank you for clarifying the design and implementation of the questionnaire. I appreciate that the survey was adapted from an existing, non-validated instrument. However, my original question remains unaddressed: how was the construct of stigma specifically operationalised in the questionnaire? The manuscript now includes a definition—"Stigma refers to how some people, conditions or places are marked out as undesirable or degraded [4-6]," Based on this definition, it is unclear whether "perception of stigma" is measured. Instead, the data may be more accurately described as participants' self-reported experiences of unfair treatment, which may or may not stem from stigma, per your definition. The ethnographic component of the study will add context to explore stigma and discrimination.

As the authors acknowledge in the text, stigma is a complex social construct; the questionnaire can certainly describe how the participants perceived being unfairly treated across several systems, but the claim that this is due to stigma is, in my opinion, an oversimplification.

VERSION 2 - AUTHOR RESPONSE

Reviewer: 2
Dr. Carla Perrotta, University College Dublin

Comments to the Author:
Thank you for clarifying the design and implementation of the questionnaire. I appreciate that the survey was adapted from an existing, non-validated instrument. However, my original question remains unaddressed: how was the construct of stigma specifically operationalised in the questionnaire? The manuscript now includes a definition—"Stigma refers to how some people, conditions or places are marked out as undesirable or degraded [4-6]," Based on this definition, it is unclear whether "perception of stigma" is measured. Instead, the data may be more accurately described as participants' self-reported experiences of unfair treatment, which may or may not stem from stigma, per your definition. The ethnographic component of the study will add context to explore stigma and discrimination.

As the authors acknowledge in the text, stigma is a complex social construct; the questionnaire can certainly describe how the participants perceived being unfairly treated across several systems, but the claim that this is due to stigma is, in my opinion, an oversimplification.

We acknowledge the importance of your point, of how unfair treatment is potentially distinct from an experience of discrimination. However, our approach of using 'unfair treatment' follows well established precedent in survey research on discrimination. The questionnaire was developed from an existing non-validated instrument, but also included detail from validated instruments. We built particularly on Gabbidon et al, who in turn follow the Minor and Major experiences of discrimination scale (see https://scholar.harvard.edu/davidrwilliams/node/32397). Whilst a question on 'unfair treatment' alone risks over simplification, we consider the following exploration of how this is attributed to particular identities and social categories grounds the experience of unfair treatment in what is potentially stigmatised, and so making this discriminatory.

We recognise though that our account of how these constructs are operationalised needed to be clearer. We have edited the text as follows, through clarifying more how our definitions are operationalised through these constructs (also in track changes through the manuscript).

"The measures we report in this paper are experiences of discrimination and their attributions, current health conditions, and demographic characteristics, detailed below.

Experiences of discrimination across multiple system domains and their attributions
Our questions about experiences of stigma and discrimination built on the Major Experiences of Discrimination Scale and Everyday Experiences of Discrimination Scale as adapted to the UK and mental health by Gabbidon et al (2014) [21]. Gabbidon et al use questions to explore the experience of discrimination – defined above as the differential treatment of the stigmatised - following the formulation of having experienced 'unfair treatment' and relate this to different settings. Respondents are then asked to attribute these experiences to demographic and social categories; such categories being what in our study we see as potentially stigmatised.

We developed a list of settings where people might report unfair treatment. We borrowed from the list of Gabbidon et al and combined this with the system settings from the original LSUC survey, settings from other scales used to understand mental health and drug use stigma [22, 24], our ethnographic data and consultations with people experiencing homelessness. We identified 18 different settings where people experiencing homelessness could experience unfair treatment. Respondents were then asked to respond to a series of statements relating to each system setting, e.g. 'I have been unfairly treated at the GP', with options of yes, no, not sure, not applicable. For analysis we also grouped these individual system and public settings into eight broader domains: housing, health, drug and alcohol services, legal services, welfare,

public settings, work and education (not all of the domains including multiple services and settings, e.g. drug and alcohol services). "
